# The Impact of Network Embeddedness on the Innovation Performance of New Generation of Employees in the Post-COVID-19 Era—The Mediating Role of Psychological Contract

**DOI:** 10.3389/fpsyg.2022.737945

**Published:** 2022-02-08

**Authors:** Jianhua Wang, Junwei Ma, Yongzhou Li

**Affiliations:** ^1^Evergrande School of Management, Wuhan University of Science and Technology, Wuhan, China; ^2^Changshu Institute of Technology, Suzhou, China

**Keywords:** network embeddedness, psychological contract, innovation performance, mediating effect, new generation of employees

## Abstract

The innovation activities of new generation of employees have the characteristics of double network embeddedness, and the degree of psychological contract fulfilment is an important factor that affects their innovation performance. Based on the attributes of internal network embeddedness and external network embeddedness, this paper builds a hypothesis model of the relationship between network embeddedness, psychological contract and innovation performance. It explores the impact and mechanism of network embeddedness on the innovation performance of new generation of employees and the mediating role of the psychological contract. Empirical research shows that network embeddedness has a positive effect on the innovation performance of new generation of employees. The psychological contract has a mediating role in network embeddedness on innovation performance of new generation of employees. These conclusions continue and deepen the research on network embeddedness and innovation performance and further enrich and expand the application of social networks in the research of individual innovation performance of new generation of employees.

## Introduction

As the micro-foundation of the innovation system, individual innovation has a huge impact on forming organizational innovation atmosphere and sustainable development. Researchers have become increasingly interested in age-related constructs other than chronological age, which has been found to explain only small amounts of variance in many important work outcomes (Rudolph et al., 2019). Generations is everywhere, generational differences have been used to describe a number of work-related phenomena (Rudolph et al., 2020). The relationships between generational membership and work-related outcomes are different from moderate to small (Costanza et al., 2012). New generation of employees has become more and more important in today’s workplace with their high level of knowledge, excellent professional skills, awareness and a strong willingness to innovate (Xiao, 2018). The innovation activities of new generation of employees require frequent exchanges and interactions with various social subjects in the internal and external environment, which in turn gives birth to close relationships with them. Those kinds of compatible relationships gradually form social networks with “embedded” characteristics and goal commonality and resource complementarity. New generation of employees is network members in the organization’s internal and external networks, and their connections with other network members will affect their ability to obtain resources. Social network theory believes that the communication and connection between network members will form different connection strengths, and the different connection strengths will affect the creation, acquisition and transmission of knowledge. Social networks provide opportunities and conveniences for the innovation activities of new generation of employees (Phelps et al., 2012). They can use their network location, information and communication technologies to gain information, control advantages and new alternatives (Reyes-Menéndez et al., 2018). With the increase in the scale and density of network connections, new generation of employees can get more substantial support such as resources and services. These advantages and support promote new generation of employees to have a confident mental state, firm belief in innovation and active work enthusiasm. At the same time, high-intensity, high-quality, and continuous network embeddedness can enhance the trust and collaboration between new generation of employees and other network members (Wang and Kim, 2012). These are conducive to new generation of employees’ creativity and innovation performance. Although new generation of employees has a high willingness to innovate, they have poor psychological endurance and unstable or unsustainable innovation performance. New generation of employees generally has a strong sense of self and pay attention to the realization of self-worth (Hou et al., 2014). Their unique social values, work values, and personality characteristics have also brought managers challenges to carry out their works (Liu and Mao, 2015). Therefore, the persistence of innovation performance also needs to play the mediating role of the psychological contract. Extant research has confirmed a significant correlation between psychological contracts and employees’ contribution to the organization (Satsomboon and Pruetipibultham, 2014).

Scholars mainly define new generation of employees from the perspective of birth time and growth background. There is currently no uniform definition of the age of the new generation. Scholars often use the Y generation as the name in the research on new generation of employees, which refers to the young group who were born after 1980 and gradually entered the labor market. The theoretically consistent view is that new generation of employees is the generation that has grown up with Internet information technology and economic globalization. They are a group with different personality characteristics, unique work values and behavior styles that are different from other generations of employees. This study defines new generation of employees as young people who were born after 1980 and have entered the workplace, including “post-80s,” “post-90s” and “post-00s,” etc. They have obvious characteristics of “Internet natives,” and they value income, self-centered, pursue freedom and equality, care about personal growth, and dare to accept challenges. Extant research on innovation performance has primarily focused on the influencing factors of innovation performance based on the organizational and individual levels. First, from an organizational perspective, scholars have studied leadership styles and methods (Kundu et al., 2019; Shafique et al., 2020), organizational support (Elsouk et al., 2021), management incentives (Wang et al., 2018), innovation atmosphere (Vessey et al., 2014), organizational environment (Lee et al., 2011; Kalyar et al., 2020) and other influencing factors. Second, from the perspective of the individual level, scholars have studied individual characteristics (Robbins, 2013), motivation (Twenge et al., 2010; Schoen, 2015), emotions (Lin et al., 2014), psychology (Erhan and Mustafa, 2018; Maia and Bastos, 2019) and other influencing factors. In addition to the factors of individuals and organizations, a new research perspective of social capital and network embeddedness has emerged in recent years to explore the impact of social interactions in complex and profound social networks on the innovation performance of organizations and their employees. From the perspective of organizational embeddedness, Gebreeyesus and Mohnen (2013) proposed that the strong connection among network members in the social networks helps high-tech enterprises to form common attitudes, propositions and beliefs, thereby promoting their innovation performance. Some scholars explored the impact of embeddedness on individual innovation performance from the perspective of organization and work embeddedness. Rass et al. (2013) believed that network embeddedness had an important influence on employees’ access to cutting-edge industry information. Gonzalez-Brambila et al. (2013) discussed the synchronization effect of the different dimensions of network embeddedness on the individual performance. Guan and Liu (2016) found that the different network positions and relationships of employees in social networks further brought about different knowledge power and social capital, thereby affecting the innovation performance of employees. With open innovation and agglomeration innovation becoming the mainstream, significant progress has been made in theoretical research and management practices related to network embeddednes and its impact on innovation performance, which provides a good literature and theoretical basis for this study.

However, extant research objects about employees’ innovation performance are mainly concentrated on people with typical job characteristics and professional positions (such as scientific research personnel, service personnel, management personnel, etc.) (Si et al., 2011; Leticia Santos-Vijande et al., 2016; Ali et al., 2020), and there are relatively few studies on different generations, especially new generation of employees. Even the existing researches on the innovation performance of new generation of employees are also more from the perspective of personnel management, work values (Hou et al., 2014; Zhu et al., 2016), identity perception (Yu, 2014), work engagement (Jing and Liu, 2018), psychological contract (Lub et al., 2011), organizational support (Zhang et al., 2012) and other predictor variables. From the perspective of network embeddedness, there is a relatively lack of relevant research on job embeddedness, organizational embeddedness, occupational embeddedness and its influence on new generation of employees’ individual behavior, personality psychology and innovation performance.

The COVID-19 pandemic has a “double-edged sword effect” on the organization’s harmonious employment relationship, which are a severe challenge and an opportunity for reconstruction. On the one hand, the epidemic will prompt organizations to make major adjustments in their business strategies. These changes will inevitably have a huge negative effect on the labor contract system and the economic rights of employees, and then affect the fulfillment of transactional psychological contracts; on the other hand, the crisis brought by the epidemic highlights many problems such as the lack of the labor dispute mediation system, union construction, and collective negotiation within the organization. These issues will surely reshape both employers and employees’ cognition and provide space for reconstructing psychological contracts (Kochan et al., 2019). In addition to the impact on the organization, the COVID-19 pandemic has also brought harm and uncertainty to employees. Employees will have varying degrees of negative emotion under the influence of the epidemic. The appearance of these negative emotions will further trigger many negative consequences, for example, job burnout and aggressive behavior (Gordijn et al., 2006). Due to the long duration of the COVID-19 pandemic, negative emotions may also evolve into negative moods and become a continuous psychological state, which is not conducive to people’s correct response to work. Studies have paid attention to the negative emotions in the population at the beginning of the COVID-19 pandemic and found that the levels of worry, fear, and anxiety are very high (Lee et al., 2020). Based on generational differences, new generation of employees who generally have poor psychological endurance and self-control ability may be more adversely affected by the epidemic.

Consistent with the previous discussion, this study intends to use the relevant theories of social network embeddedness and psychological contract, and select new generation of employees with heterogeneous characteristics such as high innovative information needs and double network embeddedness as the research objects, and use empirical research to explore the relationship and mechanism of social network embeddedness, psychological contract and innovation performance. We believe that focusing on the self-shaping of new generation of employees will develop the value hidden in their positive psychology. In turn, the functional utility of social networks and the invisible role of positive psychology will enhance their sense of responsibility and emotional investment in the organization (Hirst et al., 2009) and reduce the inconsistency of psychological contract perception (Taegoo et al., 2018), which will affect their innovative attitudes and behaviors, and ultimately promote their innovation performance.

With drastic changes in employment relationships and frequent violations of employees’ psychological contracts, human resource management in the post-epidemic era faces tremendous challenges. Therefore, in the post-epidemic era, from the perspective of social network embeddedness and psychological contract, give full use of the social network to provide new generation of employees with necessary knowledge and resources for their innovative activities and to meet their psychological and emotional needs has important practical significance. Different from previous studies, the contributions of this study are as follows: First, the research object of this study is individuals, not organizations and teams. This study takes new generation of employees, a typical heterogeneous human capital, as the research object and uses theories of network embeddedness, psychological contract, and heterogeneous human capital to explore their structural, relationship embeddedness characters and unique psychological needs. The second is that this study uses a comprehensive perspective of social capital and psychological capital to explain the innovation performance of human capital. The third is to analyze the mediating role of the psychological contract in the relationship between network embeddedness and innovation performance.

The following section presents the relevant literature review and forms hypotheses for testing. The methodology of testing the hypotheses is outlined, followed by the analysis of the results. Discussion and implications for the literature and conclusion are provided in the final section.

## Literature Review

### Network Embeddedness and the Psychological Contract

Network embeddedness refers to the behaviour of actors entering the network by establishing connections with network nodes. It is also a form in which the actors’ social relations affect their economic behaviour. Granovetter (1985) divided network embeddedness into two dimensions: structural embeddedness and relationship embeddedness. Structural embeddedness is a physical feature formed by interactive subjects in social networks, including the subject’s position in the network, network size, network stability and network density (Inkpen and Tsang, 2005). Relationship embeddedness is a bilateral relationship based on the expectation of reciprocity between interactive subjects in a social network. It mainly describes the trust relationship, cooperative relationship and degree of reciprocity between interactive subjects (Dhanardj et al., 2004). The size and structure of the network embedded by the actors and their locations in the network determine whether the actors can gather a large number of resources. At the same time, the strength and quality of the trust and sharing relationship between actors and other subjects in the social network determine the diversity and the efficiency of transmission of innovative elements such as knowledge and information to a large extent.

The psychological contract is the expectation of both parties (organisation and employees) for mutual responsibilities and obligations. As an implicit expectation, the psychological contract is widely present in the behavioural activities of the individual. Macneil (1985) divided the psychological contract into two types: transactional psychological contract and the relational psychological contract. Transactional psychological contract emphasises short-term material exchanges and mainly focuses on economic and direct returns; relational psychological contract emphasises long-term, non-material exchanges and mainly focuses on emotional and open commitments (Rousseau, 2000). Many studies agree that social network is an important factor that affects and determines the formation and development of individual psychological contracts (Han et al., 2012). Social networks provide fertile ground for understanding individual cognition (Kathryn, 2009). Kingshott and Pecotich (2007) highlighted the significance of social exchange in helping to explain the trust and commitment between organization and employees. Social networks can provide an employee with useful resources about opportunities and choices (Soojin et al., 2009). Employees’ resources affect and shape psychological contract breach perceptions (Robinson and Morrison, 2000). Psychological contract breach leads to increased organizational cynicism and lower affective, normative and continuance commitment (Lapointe et al., 2020). This study believes that due to the heterogeneity of network embeddedness degree, network status, network location and the differences of network scale, density, openness, the impact of network embeddedness on the psychological contract perception of new generation of employees is significantly different. This impact of social network on psychological contract through social interaction varies significantly depending on the “communication satisfy” (Kingshott and Pecotich, 2007). New generation of employees at the centre of the social networks and who possess rich structural holes are more likely to enhance the efficiency of psychological contracts and generate more positive psychological emotions. As such, the following hypothesis is offered:

H1: Network embeddedness has a significant positive impact on the psychological contract of new generation of employees.

H1a: Relationship embeddedness has a significant positive impact on the transactional psychological contract of new generation of employees.

H1b: Relationship embeddedness has a significant positive impact on the relational psychological contract of new generation of employees.

H1c: Structural embeddedness has a significant positive impact on the transactional psychological contract of new generation of employees.

H1d: Structural embeddedness has a significant positive impact on the relational psychological contract of new generation of employees.

### Psychological Contract and Innovation Performance

Innovation performance is a comprehensive reflection of innovation willingness, innovation behaviour and innovation results. Evaluating individual employees’ innovation performance should be a simple evaluation of rigid output indicators and the dynamic behaviour that promotes the realisation of innovation goals. Therefore, innovation performance is the unity of process innovation and result innovation (Loch and Tapper, 2002). The process innovation is usually understood as the behavioural process of innovation motivation, creative execution and realisation, mainly composed of proposing new technologies, summarising work, and bringing up new ideas (Thomas, 2010). The result innovation mainly refers to innovation itself, namely the efforts and achievements or actual items made to achieve innovation (Cooper, 1994).

In recent years, scholars focus on analysing the positive and negative effects of the fulfilment and violation of psychological contracts on employees’ innovative attitude, innovation willingness, innovative behaviour and innovation efficiency. The volume of psychological contract fulfillment is important in predicting feelings of psychological contract violation and intentions to engage in development activities (Marcelus et al., 2018). Employees perceiving a breach of the expected obligations from the employer may become unsatisfied at work (Merkouche et al., 2017), which is not helpful to the formation of trust and loyalty (Wang, 2017). Increase or decrease in organizational trust and loyalty can influence psychological contract violation, affective commitment and work engagement (Saman and Hazrati, 2017). Work engagement shows a strong association with performance. A strong organizational commitment and a high work engagement have been frequently labeled as critical success factors to achieve higher performance (Cesario and Chambel, 2017). A statistically significant negative correlation was found between psychological contract breach and employee performance (Erhan and Mustafa, 2018). Employees’ psychological contract breach may generate a weak perception of the organization and organizational distrust (Mja et al., 2021). The perception of unfulfilled expectations (psychological contract breach) adversely affects employees’ perceived organizational support, which in turn affects employees’ work outcome (Gupta et al., 2016). Psychological contract would be a significant step on the ladder of job performance (Maia and Bastos, 2019). Both transactional and relational psychological contract fulfillment can directly affect employees’ task performance (Liu et al., 2020).

As heterogeneous human capital with network embeddedness and high demand for innovative information, new generation of employees often engage in creative work in a volatile and uncertain environment. They have a high achievement orientation (Twenge et al., 2010). This situation leads them to be very interested in innovative elements, knowledge sharing, and a good atmosphere of cooperation (Hernaus and Vokic, 2014). Therefore, new generation of employees has higher psychological expectations for interpersonal relationships, organisational compatibility and comfort, knowledge and skills, and a growth environment (Fang et al., 2020). New generation of employees with a clear career plan and good psychological cognition attach great importance to fulfilling the psychological contract (Shri, 2011). This study believes that psychological contract fulfilment can reduce new generation of employees’ negative behaviours and significantly perceive the organisation’s attention and trust. Thereby, new generation of employees who are not being slack in work can put in more innovation efforts and innovation enthusiasm to obtain better innovation performance. As such, the following hypothesis is offered:

H2: Psychological contract has a significant positive impact on the innovation performance of new generation of employees.

H2a: Transactional psychological contract has a significant positive impact on the process innovation of new generation of employees.

H2b: Transactional psychological contract has a significant positive impact on the result innovation of new generation of employees.

H2c: Relational psychological contract has a significant positive impact on the process innovation of new generation of employees.

H2d: Relational psychological contract has a significant positive impact on the result innovation of new generation of employees.

### The Mediating Role of Psychological Contract

The psychological contract is usually used as a mediator variable between social environment and individual or organisational performance in the extant literature. Social interaction and sharing behaviors among network members (Yen et al., 2015; Zimmermann et al., 2017; Liu and Tang, 2020), and shared vision and cultural values (Shih and Chen, 2011; Ling et al., 2017) help increase employees’ understanding and perception of mutual obligations in employee-organizational relationships (Sharon and Mark, 2011). This improves the satisfaction of employees’ external needs and their psychological identity, and promotes the establishment and development of transactional and relational psychological contracts. Along with the fulfillment of transactional and relational psychological contracts, employees show stronger adaptability and resistance to stress in a mutually trusting environment, which in turn brings them higher innovative emotions and innovative behaviors (Kim, 2014; Fainshmidt and Frazier, 2017). At the same time, the self-drive of relational and transactional psychological contracts enables employees to make full use of various social networks, obtain more innovation resources and active psychological states, improve their creativity (Jiao et al., 2019) and strengthen the gain effect of network embeddedness on individual innovation performance. Under these circumstances, employees’ work performance is prompted and sustained by embedding engagement (Sharon and Mark, 2011). According to the theory of social exchange, when employees think that their contribution to the organization is not equal to the rewards given by the organization, the employee’s job satisfaction will be greatly reduced, and transactional psychological contract breach are likely to occur. Psychological contract breach can lead to employees’ negative behaviors (Rozhan et al., 2005; Blau and Thomas, 2009), and significantly negatively affect their performance (Restubog et al., 2006). Organizational embeddedness determines whether psychological contract breach relates to employees’ more or less innovation (Kiazad et al., 2014). Employees are most likely to reduce their work effort when they perceive that the organization has intentionally failed to live up to its commitments (Turnley et al., 2003).

New generation of employees will inevitably have relationships with enterprises, users, governments, and intermediary organisations in their innovative activities. This deep-level social network interaction enables them to reduce cognitive distance and ease negative emotions and behaviours (Parida and Örtqvist, 2015). As the size of the social network increases, employees’ well-being will increase significantly (Cheon and Park, 2019). Under positive emotional engagement and the creative atmosphere, the psychological contract of new generation of employees has been continuously verified. New generation of employees has more and more equitable psychological contracts. Equitable psychological contracts have an positive impact on job satisfaction and intrinsic motivation (Akmal and Azliyanti, 2019). Therefore, network embeddedness impacts the acquisition, integration and transformation of innovative elements of new generation of employees, which is of great significance to the construction and development of innovation knowledge system (Liu and Tang, 2020). Meanwhile, network embeddedness has an impact on the psychological support for innovation, and innovation process and innovation result through psychological contract resonance. As such, the following hypothesis is offered:

H3: Psychological contract plays a mediating role in the influence of network embeddedness on innovation performance.

**FIGURE 1:**
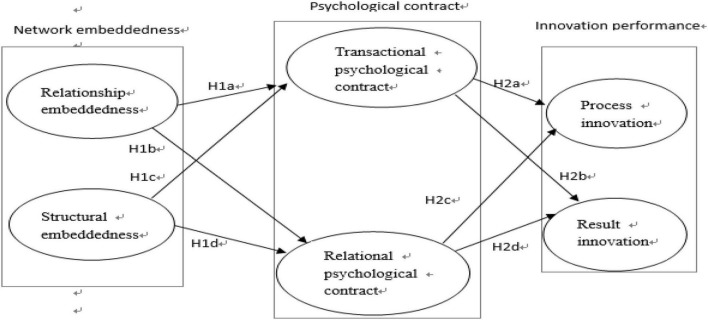
Research model.

Based on previous research, we suggest differences in different network embeddedness modes on individual innovation performance. Therefore, this study examines the impact of network embeddedness (relationship embeddedness and structural embeddedness) on innovation performance (process innovation and result innovation). Moreover, this study also examines the mediating effects of psychological contracts (transactional psychological contract and relational psychological contract) on the relationship between network embeddedness and innovation performance. Figure 1 shows the model of this study.

## Materials and Methods

### Sample and Data Collection Procedure

The questionnaire presents the statements regarding the network embeddedness, psychological contract and innovation performance. The context of the study is the manufacturing enterprises in southeastern China. Manufacturing enterprises are important because the performance improvements in this sectors are directly associated with the innovation of China (Geng et al., 2020). The settings of manufacturing enterprises are the imperative context of the study for analysis of network embeddedness and performance. Additionally, new generation of employees is becoming more and more important in the workplace and the main force of innovation (Xiao, 2018). Their innovation becomes more essential in this setting.

To understand the proposed relationships, we collected data from manufacturing enterprises in China. Before distributing the questionnaire, a pilot test was conducted with 60 participants. As a result, a few minor changes were made to improve the clarity and complete the initial questionnaire. First, through factor analysis, the factors with their eigenvalue greater than one are extracted. Second, according to the feedback in the pilot test, the language expression of some measurement items was corrected, and some items that did not conform to the actual situation were deleted. After ensuring that there are no problems, the final survey was undertaken. These questionnaires are collected through a self-administered online questionnaire, with the dissemination of the questionnaire to a convenient sample by e-mail with a QR code or link. For item rating, a 5-Likert-scale was used in the survey. This approach is the most recommended for online surveys, as it offers ease of response and helps collect information about the intensity of the participants’ feelings (Reyes-Menendez et al., 2019a). A random sampling was employed in this survey. This survey was undertaken in different periods of the day (mornings, afternoons, and evenings) throughout one month to capture different cohorts of respondents. The respondents were required to answer questions objectively and fairly based on the facts. Of 300 questionnaires distributed, a total of 242 fully completed and useable questionnaires were generated.

As shown in Table 1, among the 242 new generation of employees, over half of the respondents (62%) are male. Most respondents have university degrees. The age groups are rather evenly distributed. Most of the respondents have a tenure of 1–5 years (75.2%), about 24.8% of the respondents have accumulated work experience of more than five years.

**TABLE 1:** Demographics of the survey respondents.

Variable	N	Percentage
Male	150	62.0%
Female	92	38.0%
Less than 26	25	10.3%
Between 26 and 30	68	28.1%
Between 31 and 35	95	39.3%
Between 35 and 40	54	22.3%
High School or less	35	14.5%
Bachelor degree	128	52.9%
Graduate degree	79	32.6%
Less than one year	98	40.5%
Between 2 and 5 years	84	34.7%
Between 6 and 10 years	60	24.8%

### Measures

Related variables are measured on a 5-Likert-scale from “strongly disagree” to “strongly agree.” The measurement of each variable and the design of the questionnaire are explained below.

#### Network Embeddedness

According to Granovetter (1985), network embeddedness is measured from two dimensions: structural embeddedness and relationship embeddedness. The five-item scale directly measures the four characteristics of structural embeddedness: location, scale, density and stability (Bart et al., 2010). The four-item scale is used to measure relationship embeddedness in terms of the trust, information sharing, relationship strength and relationship quality (Simpson, 2015).

#### Psychological Contract

Concerning Ahmad and Zafar (2018), the psychological contract is measured from two dimensions: transactional and relational. The transactional psychological contract is mainly described from responsibility, material incentives, and economic benefits, with a five-item scale. The relational psychological contract is mainly described from relationship status, organisational support and development opportunities, with a six-item scale.

#### Innovation Performance

Based on Hogan et al. (2011), innovation performance was measured from the two dimensions of process innovation and result innovation. Process innovation is mainly described from innovation ability, innovative ideas, with a five-item scale. Result innovation is mainly described from the aspects of innovation output such as products or services, technologies, methods or procedures, with a five-item scale.

## Data Analysis and Results

### Reliability and Validity Analysis

Use SPSS20.0 for reliability analysis and factor analysis. The results show that Cronbach’s alpha values of relationship embeddedness, structural embeddedness, relational psychological contract, and result innovation are all higher than 0.9. The Cronbach’s alpha values of transactional psychological contract and process innovation are between 0.8 and 0.9, indicating acceptable reliability. The KMO value of each scale is greater than 0.7, and each scale has passed Bartlett’s test, indicating good validity. This study further uses AMOS24.0 to conduct confirmatory factor analysis to calculate the fitness index values of the three variables of network embeddedness, psychological contract, and innovation performance. From the results in Table 2, it can be seen that the fitness index value of each CFA is within the range of the reference standard, indicating a good fitting effect. The standard factor loadings are all over 0.5, the composite reliabilities (CR) are all over 0.7, and the average variance extracted (AVE) are all over 0.5, indicating good convergence validity.

**TABLE 2:** Results of confirmatory factor analysis.

Construct				Convergent validity		Fit index				
		Item	Fl	CR	AVE	χ2/df	CFI	TLI	RMSEA	RMR
Network embeddedness	Relationship embeddedness	RE1	0.884	0.909	0.716	2.384	0.952	0.934	0.057	0.028
		RE2	0.862							
		RE3	0.782							
		RE4	0.853							
	Structural embeddedness	SE1	0.814	0.916	0.685					
		SE2	0.842							
		SE3	0.781							
		SE4	0.832							
		SE5	0.866							
Psychological contract	Transactional psychological contract	JY1	0.732	0.873	0.580	2.601	0.915	0.932	0.065	0.034
		JY2	0.776							
		JY3	0.764							
		JY4	0.772							
		JY5	0.762							
	Relational psychological contract	GX1	0.802	0.913	0.638					
		GX2	0.767							
		GX3	0.804							
		GX4	0.777							
		GX5	0.793							
		GX6	0.845							
Innovation performance	Process innovation	IW1	0.815	0.896	0.634	2.971	0.906	0.956	0.069	0.038
		IW2	0.790							
		IW3	0.787							
		IW4	0.753							
		IW5	0.833							
	Result innovation	IP1	0.852	0.912	0.675					
		IP2	0.844							
		IP3	0.767							
		IP4	0.837							
		IP5	0.805							
Standard		> 0.7		>0.7	> 0.5	< 3	> 0.9	>0.9	< 0.08	<0.05

In Table 3, it can be seen that the square root of AVE is greater than the correlation coefficient, which meets the discriminative validity criterion, indicating good discriminative validity. The variance inflation factor (VIF) values of relationship embeddedness, structural embeddedness, transactional psychological contract, and relational psychological contract, respectively, are 3.943, 3.201, 2.908, and 2.674, which excludes the possibility of multicollinearity among the independent variables.

**TABLE 3:** Correlations and square root of AVE (diagonal).

Variable	1	2	3	4	5	6
Relationship embeddedness	0.846					
Structural embeddedness	0.619**	0.828				
Transactional psychological contract	0.654**	0.559**	0.762			
Relational psychological contract	0.600**	0.583**	0.481**	0.799		
Process innovation	0.494**	0.455**	0.417**	0.456**	0.796	
Result innovation	0.415**	0.594**	0.571**	0.528**	0.560**	0.822

** Correlation is significant at the 0.01 level (2-tailed). The figures on the diagonal are the square root of AVE, and the rest are the correlation coefficients of the latent variables.

To further test the discriminative validity, this study uses a single group to generate two models (namely the unconstrained model and the constrained model) and then compare the chi-square value difference of the two models. If the chi-square value difference is large enough and reach a significant level (*p* < 0.05, CMIN Difference = 3.841; *P* < 0.01, CMIN Difference = 6.635; *P* < 0.001, CMIN Difference = 7.879), it shows that there is a significant difference between the two models, and the two constructs have discriminative validity. The discriminative validity tests of the pairwise latent variables are carried out, respectively. According to the results, the chi-square value in the unconstrained model is significantly reduced than in the constrained model. The chi-square difference is greater than 7.879, indicating good discriminant validity. This result further proves that there is good discriminant validity among six variables.

### Common Method Bias Analysis

We assessed common method bias in two ways. First, we perform exploratory factor analysis on all items (Harman single factor test). When the factors are not rotated, the variance contribution rate of the first factor is 37.831%. It is less than the threshold of 40%, indicating that there is no serious common method bias problem in the sample. Second, we use the latent error variable control method to test the common method bias (Podsakoff et al., 2003). Concerning Harmon’s single factor test, 30 items in the three latent variables of network embeddedness, psychological contract and innovation performance are loaded onto a single latent factor (the covariant factor CMV) to establish a single-factor model. According to the dimensionality of variables in the scale, a five-factor model consisting of relationship embeddedness, structural embeddedness, psychological contract, process innovation, and result innovation is established. Then, the covariant factor is added to the five-factor model to form a six-factor model. The fitness index of each model is shown in Table 4.

**TABLE 4:** Common method bias test.

Model	Factor	Fitness index					
		χ2/df	CFI	TLI	GFI	RMSEA	RMR
Single-factor model	CMV	1.961	0.867	0.857	0.690	0.097	0.118
Five-factor model	RE;SE;JY+ GX;PI;RI	2.018	0.902	0.898	0.885	0.080	0.036
Six-factor model	RE;SE;JY+ GX;PI;RI;CMV	2.025	0.861	0.847	0.677	0.256	0.073

*RE = Relationship Embeddedness, SE = Structural Embeddedness, JY = Transactional Psychological Contract, GX = Relational Psychological Contract, PI = Process Innovation, RI = Result Innovation, CMV = Common Method Variation(Covariant factor), “+” means factor combination.*

The results demonstrate that the single-factor model fit poorly to the data. That is, all the items do not belong to the same variable. The fit indices of the five-factor model are better than that of the single-factor and six-factor models. It provides further evidence that common method bias does not appear to be a serious problem in this study and does not influence the significance of the results (Olson et al., 2005).

### Path Analysis and Hypotheses Testing

This study builds the relationship between network embeddedness and psychological contract (model 1) and the relationship between psychological contract and innovation performance (model 2). Table 5 shows the fitness index values of each model. The results show that the models have acceptable fit indices.

**TABLE 5:** Comparison of the models.

Model	Fit indices					
	^2^/d*f*	CFI	TLI	GFI	NFI	RMR
Model 1	2.223	0.937	0.915	0.859	0.913	0.038
Model 2	2.450	0.859	0.937	0.877	0.934	0.038

Table 6 shows the coefficient, standard error, critical ratio (C.R.) and significance level of each model. The results show that relationship embeddedness has a significant impact on transactional psychological contract (β = 0.643, *P* < 0.001) and relational psychological contract (β = 0.733, *P* < 0.001). Structural embeddedness has a significant impact on transactional psychological contract (β = 0.321, *P* < 0.001) and relational psychological contract (β = 0.232, *P* < 0.001). Transactional psychological contract has a significant impact on process innovation (β = 0.256, *P* = 0.002) and result innovation (β = 0.246, *P* = 0.006). Relational psychological contract has a significant impact on process innovation (β = 0.685, *P* < 0.001) and result innovation (β = 0.670, *P* < 0.001). Therefore, H1a, H1b, H1c, H1d, H2a, H2b, H2c, H2d are supported.

**TABLE 6:** Results of path analysis.

Path relationship	Estimate	C.R.	P	Support
Relationship embeddedness → Transactional psychological contract	0.643	6.914	***	H1aYes
Relationship embeddedness → Relational psychological contract	0.733	8.236	***	H1bYes
Structural embeddedness → Transactional psychological contract	0.321	3.527	***	H1cYes
Structural embeddedness → Relational psychological contract	0.232	4.549	***	H1dYes
Transactional psychological contract → Process innovation	0.256	3.084	0.002	H2aYes
Transactional psychological contract → Result innovation	0.246	2.828	0.006	H2bYes
Relational psychological contract → Process innovation	0.685	7.287	***	H2cYes
Relational psychological contract → Result innovation	0.670	8.171	***	H2dYes

*** means 1% significance level.

Next, we examine psychological contract’s mediating effects. The premise of the mediating effect is that the independent variable is significantly correlated with the dependent variable; otherwise, the mediating variable will not be considered (MacKinnon et al., 2002; Wen et al., 2004). This study builds the relationship between network embeddedness (relationship embeddedness and structural embeddedness) and innovation performance (process innovation and result innovation). We find that the regression coefficient of relationship embeddedness on result innovation is not significant (β = 0.124, *P* = 0.065). So we will stop testing the mediating effect of this path later in the study.

Bootstrapping is conducted to test the mediating effect of the psychological contract presented in our model. We implement bootstrapping using the Process macro from Hayes et al. (2017). The indirect effects are significantly different from zero (i.e., the mediating effects are significant) when zero is not in the confidence intervals. Results are presented in Table 7. On the research path between relationship embeddedness and process innovation, indirect effects of transactional psychological contract and relational psychological contract are 0.0693 and 0.4540. Zero is not included in these confidence intervals of indirect effects and direct effect. It confirms that transactional and relational psychological contracts have partial mediating effects on the relationship between relationship embeddedness and process innovation. Similarly, it can be seen that in the other two relationship paths, transactional psychological contract and relational psychological contract both have a full mediating effect. H3 is supported.

**TABLE 7:** Mediation analysis with bootstrap.

Research path	Point estimated value		Bias-corrected (95%)	
			Lower	Upper
Relationship embeddedness → Process innovation	Total effect	0.731	0.677	0.882
	Direct effect	0.208	0.185	0.379
	Indirect effect of transactional psychological contract	0.069	0.026	0.278
	Indirect effect of relational psychological contract	0.454	0.247	0.516
Structural embeddedness → Process innovation	Total effect	0.201	0.687	0.831
	Direct effect	–0.004	–0.035	0.303
	Indirect effect of transactional psychological contract	0.029	0.032	0.280
	Indirect effect of relational psychological contract	0.176	0.269	0.534
Structural embeddedness → Result innovation	Total effect	0.189	0.722	0.901
	Direct effect	–0.077	–0.009	0.225
	Indirect effect of transactional psychological contract	0.116	0.035	0.226
	Indirect effect of relational psychological contract	0.150	0.443	0.766

## Discussion

The study shows that network embeddedness has a significant direct effect on the psychological contract. This finding is consistent with that in Kraak and Altman (2016). The psychological contract directly affects innovation performance, consistent with Kiazad et al. (2018) and Maia and Bastos (2019). Network embeddedness has a significant direct effect on innovation performance, consistent with Tolstoy and Agndal (2010) and Shipley et al. (2013). Such consistent findings indicate that network embeddedness is critical to actively promote the germination and growth of innovation and achieve innovation performance.

However, we further find that structural embeddedness has a significant positive impact on process innovation and result innovation, while relationship embeddedness only positively impacts process innovation. Its impact on result innovation is not significant. This finding is consistent with the “relationship embeddedness paradox” (Uzzi, 1997). This phenomenon may be that network embeddedness has expanded the diverse knowledge sources of new generation of employees. Highly trustworthy and continuous relationship embeddedness can help new generation of employees grasp more opportunities to obtain innovative resources. Connections may lead to the repetition and circulation of information paths and bring about information homogeneity and redundancy. Therefore, network closure, ignoring the constantly changing product and technology requirements, is harmful to innovation performance improvement.

The psychological contract exists widely in the relationship between employees and organisations. The new generation of employees’ commitment perception and emotional input play a role in their performance with process innovation and result innovation. The positive changes in the attitude and psychological perception of new generation of employees can further stimulate their enthusiasm for innovation and enhance their innovation performance. On the contrary, the breach of the psychological contract harms innovation performance. This finding is consistent with that in Kiazad et al. (2014) and Merkouche et al. (2017), although in different study settings. The result indicates that the network embeddedness of new generation of employees with the organisation’s internal and external networks affects their psychological perception and attitudes, which effectively help the individual’s career development achieve the intrinsic “psychological contract” efficiency and their behaviour with innovation. Large-scale, diversified and strongly connected network embeddedness can help new generation of employees to form common beliefs, values, unified norms and a common understanding of things.

The mediation effect exerted by psychological contract shows the sequential effects of new generation of employees’ network embeddedness on commitment perception and emotional investment to the organisation and subsequent innovation behaviours. In particular, confirmation of a significant mediation effect reinforces the mediating role of the psychological contract. This finding conforms to that in Han et al. (2012) and Kraak and Altman (2016). At the same time, in further research, we find that transactional psychological contract plays a partial mediation effect on the relationship between new generation of employees’ relationship embeddedness and their process innovation. But it plays a full mediation effect on the relationships between structural embeddedness and process innovation, structure embeddedness and result innovation. The mediation effect of a relational psychological contract is consistent with that of a transactional psychological contract. This evidence means that the psychological contract strengthens the positive effect of structural embeddedness on process innovation and result innovation. Still, it is not enough to promote new generation of employees with relationship embeddedness to show more process innovation performance. This phenomenon may be that the mutual expectations and mutual responsibilities between employees and organisations have changed significantly with the employment relationship changes. Due to the typical personality traits and the fierce competition for survival and career development, new generation of employees with relationship embeddedness have a high sense of trust and responsibility. They suggest that the realisation of psychological contract is only the basic return of the organisation to them. High psychological contract fulfilment can only give them “no dissatisfaction” but not “complete satisfaction.” Therefore, relationship embeddedness is not enough to make new generation of employees fully stimulate their willingness to innovate.

## Implications

The results of this study provide important implications for how to motivate new generation of employees to create higher innovation performance effectively.

First, organisations where new generation of employees work must pay attention to innovation and the process factors involved in the realisation of innovation goals, actively encourage their innovative thinking, guide their innovative behaviours, and ultimately stimulate their inner innovation needs.

Second, organisations need to enhance the attractiveness of the organisation’s internal and external networks to new generation of employees and promote innovation opportunities, innovation ability and innovation motivation. It is necessary to improve the frequency and scale of communication with the network members and help new generation of employees obtain high-frequency, wide-boundary network connections and ideal network locations. Organisations can improve the quality of resource flow through high-quality social interaction between network members, establish trust mechanisms, communication mechanisms and coordination mechanisms for multiple subjects inside and outside the networks, and promote sticky and implicit knowledge acquirement, improve the familiarity, trust and mutual understanding among network members, actively build a foundation for innovation cooperation between network members, and guide the formation of a sharing mechanism that meets the needs of innovation.

Third, organisations need to improve the psychological contract’s effectiveness of new generation of employees, help them establish positive psychological emotions, strengthen innovation perception and innovation identity, and strive to stimulate their innovation willingness and vitality, and ultimately promote innovative behaviours. Organisations should increase innovation support and enhance new generation of employees’ sense of belonging. Efforts should be made to enhance their sense of innovative accomplishment by strengthening and regulating the two-way commitment between organisations and employees and establishing a good commitment recognition system about loyalty and responsibility. Organisations can offer long-term organisational care based on new generation of employees’ inner needs and psychological perceptions, which is a good way to maintain and update their transactional and relational psychological contracts.

Fourth, during the COVID-19 pandemic and the post-epidemic era, social network support for new generation of employees is particularly important. Support and trust obtained from the internal and external networks can effectively alleviate the psychological pressure of new generation of employees and respond to emergencies, and ultimately help them to avoid the impact of stressful events or be less affected when facing high work pressure, to maintain their good work performance. Therefore, organisations can take various measures to provide timely and effective resource support to employees in stressful situations to reconstruct a higher-quality psychological contract.

## Conclusion

Based on data collection of 242 new generation of employees, this study conducts a theoretical discussion and empirical test on the relationship between network embeddedness (structure embeddedness, relationship embeddedness) and new generation of employees’ innovation performance (process innovation, result innovation); It also reveals the mediation effect of psychological contract (transactional psychological contract and relational psychological contract) on their relationship. At the same time, this study empirically examines and interprets the “relationship embedding paradox” in the research of the relationship between network embeddedness and new generation of employees’ innovation performance. Studies have shown that the heterogeneous characteristics of new generation of employees determine the duality of their network embeddedness. The multiple network interactions of new generation of employees have greatly acquired more heterogeneous and richer knowledge. At the same time, in a wide range of social interactions, they have significantly increased their psychological expectations and stimulated their innovation willingness and emotional engagement in innovation, thereby enhancing innovation performance. Network embeddedness has a positive predictive effect on the psychological contract and innovation performance of new generation of employees. Network embeddedness enables new generation of employees not only to obtain professional knowledge in related fields, but also to communicate with and establish business contacts with internal and external network members of the organization, on this basis, to obtain innovation support. The relationship with the network members and the network position they occupied, the degree of trust and information sharing between members also greatly affect the innovation performance of new generation of employees. Network embeddedness can influence innovation performance through the mediation of psychological contract. That is, higher psychological contract can directly lead to better innovation performance of new generation of employees, or organizations can also improve their psychological contract level by increasing their network embeddedness to ultimately achieve the improvement of their innovation performance.

This study also gets a meaningful conclusion. This research enriches the existing literature on social capital theory, psychological capital theory, and network embeddedness mechanism by constructing a framework of network embeddedness, psychological contract and innovation performance. This research takes new generation of employees of Chinese manufacturing enterprises as the research object, and puts forward suggestions for employee management in the post-epidemic era. We find that improving the psychological contract level of new generation of employees by enhancing social exchange and social interaction may become a new way for enterprises to intervene and develop employees’ psychological contracts and improve their innovative performance. When the human resource management department of an enterprise recruits employees with a high degree of network embeddedness and positive emotions, it can reduce the cost of post-training. For employees who have already joined, the improvement of network embeddedness and psychological contract fulfillment will help improve their innovation performance. At the same time, in the post-epidemic era, organizations need to pay attention to changes in employees’ negative emotions, actively assess the long-term impact of the epidemic on the mental health of new generation of employees, propose measures to prevent or reduce the impact of the epidemic on mental health from the perspective of employee management, and increase supports for employees.

## Limitations and Future Research

Although various efforts have been taken to ensure rigorousness of this research, some limitations arise for attention and caution. First, the data comes from manufacturing enterprises in southeastern China, most of the participants have a certain degree of education, and the sample is underrepresented in the low-age groups, low-educated groups and groups of different nationalities. Therefore, the results may not be generalised to other contexts. Second, the existing data does not explore the heterogeneity of the impact of network embeddedness on their innovation performance in the post-epidemic era in different industries, different occupations, etc. Third, this study did not take into account the possible new changes in employees’ psychological emotions and behavior in the post-epidemic era.

With the changes of the epidemic, whether employees’ psychological emotions can be relieved in time or whether they will continue to develop into a negative mood still needs attention. What are the impacts of new generation of employees’ work status and employment relationship changes in the epidemic on employees’ psychological contracts? How to use psychological contracts to reduce negative emotions, alleviate work pressure, and improve work adaptability and work performance? In addition, the impact of the epidemic on economic and social life has gradually emerged with its development, and these economic and social variables have also had a huge impact on the behavior of enterprises and employers. Employers may weaken the economic rights of employees, and they may also provide timely and effective resource support to employees in a stressful situation. All these may enhance the sense of organizational identity of employers. These issues need to be studied in depth, in order to deeply explore the role of psychological contract in the relationship between network embeddedness and new generation of employees’ innovation performance.

In addition, this study only explores the impact of network embeddedness on employees’ innovation performance. Future research should explore other predictor variables related to employees’ innovation performance. For example, studies have shown that non-compliant tasks (Zhou et al., 2018; Kottwitz et al., 2019), leader interpersonal emotion management (Little et al., 2016; Niven et al., 2019) and mind maps (Reyes-Menendez et al., 2019b) have an impact on employees’ emotions and work attitudes, which in turn will affect employees’ innovation performance. Besides, this study only considers the mediating effect of psychological contract, and there may be other mediators. For example, as task goals and task complexity increase, it becomes more difficult for employees to manage their own negative emotions. At this time, employees need more emotional assistance from the internal and external networks of the organization. Therefore, future research can focus on the effects of variables such as task goals, job characteristics.

## Data Availability Statement

The original contributions presented in the study are included in the article/supplementary material, further inquiries can be directed to the corresponding author.

## Ethics Statement

The studies involving human participants were reviewed and approved by Ethics Committees of Wuhan University of Science and Technology and Changshu Institute of Technology. The patients/participants provided their written informed consent to participate in this study.

## Author Contributions

JW, JM, and YL distributed with the work done in the project. JW searched the background materials, designed the analytical characterization and empirical study frame, and made the critical revision and editing. JM analyzed the data and evaluated the results. YL analyzed concepts and perfected the framework. All authors have contributed to writing the manuscript.

## Conflict of Interest

The authors declare that the research was conducted in the absence of any commercial or financial relationships that could be construed as a potential conflict of interest.

## Publisher’s Note

All claims expressed in this article are solely those of the authors and do not necessarily represent those of their affiliated organizations, or those of the publisher, the editors and the reviewers. Any product that may be evaluated in this article, or claim that may be made by its manufacturer, is not guaranteed or endorsed by the publisher.
